# Sensory determinants of the autonomous sensory meridian response (ASMR): understanding the triggers

**DOI:** 10.7717/peerj.3846

**Published:** 2017-10-06

**Authors:** Emma L. Barratt, Charles Spence, Nick J. Davis

**Affiliations:** 1 Seaham, Sunderland, UK; 2 Department of Experimental Psychology, University of Oxford, Oxford, UK; 3 Department of Psychology, Manchester Metropolitan University, Manchester, UK

**Keywords:** ASMR, Multisensory, Misophonia, Flow state, Trigger, Synaesthesia

## Abstract

The autonomous sensory meridian response (ASMR) is an atypical sensory phenomenon involving electrostatic-like tingling sensations in response to certain sensory, primarily audio-visual, stimuli. The current study used an online questionnaire, completed by 130 people who self-reported experiencing ASMR. We aimed to extend preliminary investigations into the experience, and establish key multisensory factors contributing to the successful induction of ASMR through online media. Aspects such as timing and trigger load, atmosphere, and characteristics of ASMR content, ideal spatial distance from various types of stimuli, visual characteristics, context and use of ASMR triggers, and audio preferences are explored. Lower-pitched, complex sounds were found to be especially effective triggers, as were slow-paced, detail-focused videos. Conversely, background music inhibited the sensation for many respondents. These results will help in designing media for ASMR induction.

## Introduction

The autonomous sensory meridian response (ASMR) is a sensory phenomenon typically characterised by electrostatic-like tingling across the scalp, following the line of the spine downwards, extending to the arms and further depending on the intensity of the response (Barratt & Davis, 2015). These tingling sensations can be elicited in response to a number of auditory and visual triggers. Often accompanied by a feeling of relaxation, many individuals who experience ASMR actively engage with the sensation in order to relieve negative mood, and to induce sleep (Barratt & Davis, 2015).

It has taken some time to recognise ASMR as a distinct and atypical experience, likely due to its conflation with ‘frisson’ (Colver & El-Alayli, 2016). However initial research into ASMR has been prompted, in recent years, by the establishment of a large online community. As ASMR can be induced in a number of ways, both in everyday life and through the consumption of specific media, this community currently resides mainly on the video sharing website Youtube. Many videos on this platform intended for use in ASMR induction boast millions of views (Barratt & Davis, 2015). Many of the most popular ASMR videos include interpersonal triggers such as whispering and personal attention (e.g. haircuts), alongside more abstract, non-person-centric triggers such as crisp sounds, and focused tasks (e.g. towel folding, painting nails; Barratt & Davis, 2015). It should, though, be noted here that this list is non-exhaustive, and the effectiveness of particular triggers varies widely between ASMR capable individuals.

To date no studies exist of the prevalence of ASMR-capability in the general population, although anecdotally many researchers have observed that people are aware of the sensation, but were not aware that it had a name or that other people necessarily also experienced it. However, among those who do report ASMR, there seems to be a concordance in reported personality factors, including elevated openness (Fredborg, Clark & Smith, 2017; McErlean & Banissy, 2017), suggesting a possible predisposition among some people to seek or experience ASMR.

ASMR shares some characteristics with the state of flow, defined as a deeply immersive sense of relaxation and engagement (Csikszentmihalyi, 1979). The flow state contains two components, related to active performance, such as the feeling of operating at peak performance, and passive experience, such as the feeling of time passing at an altered rate (Jackson & Marsh, 1996). This latter component is particularly reminiscent of ASMR, with both states involving a sense of deep relaxation and of well-being, although even the passive component of flow is task-directed whereas ASMR seem to require complete passivity from the person. Previous research has indicated a link between the states (Barratt & Davis, 2015), and a better understanding of the relationship may help in promoting those elements of the flow state that are thought to be helpful in domains such as sports or education (Lee, 2005; Swann et al., 2012).

In recent years, some authors have suggested that ASMR may be related to misophonia (Schröder, Vulink & Denys, 2013; Edelstein et al., 2013), a seemingly contrasting audio-sensory experience in which triggering sounds cause outbursts of anger and disgust. The production of these emotions, produced by sounds such as chewing and breathing, are automatic, and can be severe enough to warrant psychological intervention in efforts to reduce these automatic negative reactions (Bernstein, Angell & Dehle, 2013). This experience contrasts with reports of the ASMR experience, and indeed many who suffer from misophonia also report experiencing ASMR. A recent study by Rouw & Erfanian (2017) highlighted that 50% of those suffering from misophonia involved in the research also experienced ASMR. It has been theorised that perhaps ASMR and misophonia represent opposite ends of a spectrum (Barratt & Davis, 2015) of experience. The idiosyncrasy of misophonia and ASMR is highlighted in the case of ‘mouth sounds’: for some people the sound of chewing or lip-smacking is highly relaxing, while for others it is highly aversive, and mouth sounds are frequently mentioned in studies of the two phenomena. There does not appear to be a good reason why different people should respond so differently to the same stimulus, and further study is needed to understand the affective qualities associated with stimuli that evoke ASMR or misophonia in different people.

Research into the neural correlates and mechanisms of ASMR is still in its infancy. That said, studies suggesting the presence of structural and functional variation between typical and ASMR-capable populations are now starting to appear (Smith, Fredborg & Kornelsen, 2017). For instance, the latter authors investigated the default mode network in those capable of ASMR using resting state functional magnetic resonance imaging. Their analyses revealed evidence of decreased functional connectivity, yet increased connectivity between regions of the occipital, frontal, and temporal cortices in ASMR-capable participants (*N* = 11) when compared to non-ASMR-capable matched controls. The authors posited that this was evidence of a ‘blending’ of multiple resting state networks, which may give rise to the kind of sensory experiences that come about during ASMR. In support of the view that ASMR and misophonia represent opposing ends of a spectrum, it has been suggested that misophonia is also associated with abnormal resting state or functional connectivity (De Ridder et al., 2011; Kumar et al., 2017). Alteration in the functional connectivity of the brain suggests that people with chronic conditions such as pain syndromes may use ASMR to manage brain states, hinting at an impaired ability to switch between the brain’s resting state and a more active executive state (Barratt & Davis, 2015; Goulden et al., 2014).

Previous exploration of the kinds of stimuli that trigger ASMR has largely focused on interpersonal triggers, and the content of videos featuring hosts (referred to within the ASMR community as ‘ASMRtists’), or individuals carrying out roleplay situations. This largely reflects the nature of ASMR content on the internet, where the powerful triggers of whispering and crisp sounds are incorporated into a situation that implies close contact with the ASMRtist, such as a dental examination or a beauty therapy appointment. However, this approach risks mischaracterising ASMR as a purely interpersonal experience. As a result, the qualities of effective triggers not directly related to interpersonal actions made by a host, such as the properties of effective trigger objects, have largely been overlooked.

Therefore, the current study was designed to further explore the qualities of ASMR-triggering stimuli, both with and without a host or ASMRtist, in individuals who experience the sensation. In particular, it aims to better understand those factors that allow non-interpersonal stimuli to be effective triggers, and to understand how to better design ASMR stimuli for research purposes. Using an online questionnaire, we surveyed people who self-report experiencing ASMR, and asked about the content that most reliably triggers the sensation. In this exploratory study, we wanted to gather information about the features of the objects within ASMR content, and about the atmosphere and setting of media that people interact with. We therefore explored the following themes: timing (length of time a trigger is operative) and trigger load (number of effective triggers present); atmosphere and characteristics of ASMR content; ideal spatial distance from various types of stimuli; visual characteristics, context, and use of ASMR triggers; audio preferences. Information gathered here will be of interest to creators of ASMR content, and to researchers interested in the nature of ASMR-triggering stimuli.

## Methods

### Participants and procedure

A volunteer sample was recruited via social media platforms Facebook, Reddit, and Twitter. Participants completed an online questionnaire remotely via the Qualtrics survey platform (Version 02/2017, http://www.qualtrics.com). All participants confirmed that they experienced ASMR, and data were excluded from analysis if this checkbox was not ticked. The duration of the survey from start to finish was approximately 25 min. Informed consent was obtained from all participants. Ethical approval for this study was obtained from the local ethics committee at Manchester Metropolitan University, UK (REF: HPSC-1454).

### Materials

This study used an online questionnaire. All of the questions included can be found in Appendix A. A brief description and rationale behind each section is included below for reference.

Previous research has indicated that entering a flow state is an important part of the ASMR experience (Barratt & Davis, 2015). As such, the ‘atmosphere’ section of the survey used both Likert and optional comment sections in order to gather information on the ideal atmosphere and execution of ASMR content.

Though potential ASMR triggers are seemingly idiosyncratic, with effective trigger stimuli varying widely between individuals (Barratt & Davis, 2015), a section probing preferred physical aspects of trigger objects was included. This section aimed to identify and better understand which aspects of trigger stimuli are particularly interesting to ASMR-capable viewers, so that they may be pursued in future research. Additionally, the ideal spatial distance from variously-sized trigger objects and actions was investigated, in order to gain insight into ideal camera distances from various stimuli when creating content for lab use. In order to assess whether or not triggers could be manipulated to higher inductive effectiveness when viewers had a sense of aspects other than physical properties, a short section on provision of wider context was included. We also included a section that asked respondents to imagine themselves in stereotyped situations, to understand if wider environmental cues could prompt ASMR sensations. We chose a ‘bar setting’, reflecting a situation of high sociability, and ‘Scottish countryside’, reflecting a more solitary, natural environment.

### Data analysis

Due to the length of the questionnaire, and therefore likely high drop-out rate, not all questions were forced. This allowed the participants to skip some sections which may not be relevant to their ASMR experience. Participants who skipped large portions of the survey were removed from the data set. *N* for each section is reported alongside results. Where comparisons of mean have been conducted, all participants included in analyses responded to both variables. All analyses were carried out in SPSS and Microsoft Excel.

## Results

### Demographic information

A total of 130 participants (male = 33, female = 91, transgender = 2, non-binary = 4) from across the globe volunteered their responses. The average age of the sample was 35 years old (SD = 9.2 years, range = 25–65). The majority of the sample (53%) was located in the United States of America, with other popular locations being the UK (20%), mainland Europe (10%), and Canada (9%).

All of the participants self-reported as experiencing ASMR. About 43% of the sample confirmed that they also experience misophonia, 17% responding that they were unsure whether or not they had the condition.

### Timing and trigger load

Participants (*N* = 127) most often reported that their favourite online ASMR content focused on particular triggers lasting for between 1 and 5 min (38%), or 6–10 min (30%). About 15% of the sample favoured focus on a particular trigger to last between 11 and 20 min, and a further 15% identified the ideal duration to be 21 min or longer.

The data showed some variation in the number of triggers that participants felt comfortable focusing on at once. Nearly half the respondents (47%) reported two triggers as optimal. About 24% prefer one trigger at a time, 15% favour three triggers, and the remaining 13% prefer four or more at once.

### Atmosphere

**Figure 1:**
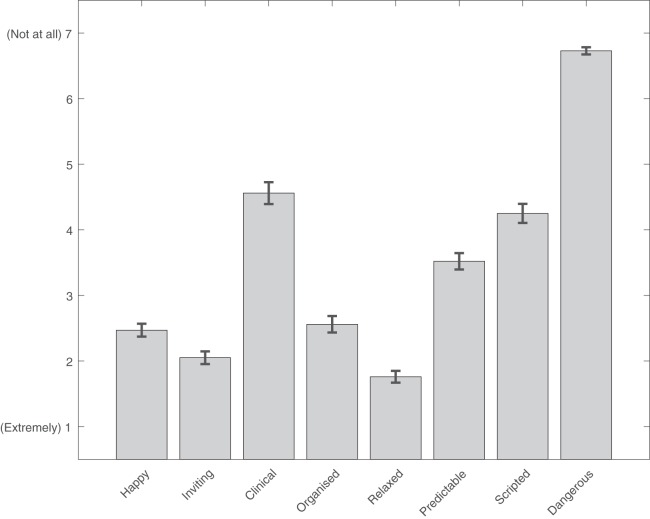
Characteristics associated with effective ASMR media. Ratings of characteristics associated with an effective ASMR video. Lower ratings mean a greater association.

Participants (*N* = 124) rated the atmosphere of their favourite ASMR video on a scale of 1 (extremely) to 7 (not at all) according to the following adjectives: happy (*M* = 2.47, SD = 1.09), inviting (*M* = 2.05, SD = 1.07), clinical (*M* = 4.56, SD = 1.86), organised (*M* = 2.56, SD = 1.40), relaxed (*M* = 1.76, SD = 1.00), predictable (*M* = 3.52, SD = 1.39), scripted (*M* = 4.25, SD = 1.62), and dangerous (*M* = 6.73, SD = 0.61; see Fig. 1).

Very few participants (*N* = 13) chose to respond to a section involving imagining achieving ASMR in a bar setting. Of those who completed the relevant Likert scale, similar to that above, assessing the qualities of a bar that would facilitate ASMR, similar results emerged. Participants favoured a welcoming, warm-looking (*M* = 2.96, SD = 1.17), happy (*M* = 2.96, SD = 1.19), and inviting (*M* = 2.86, SD = 1.06) environment. Atmospheric and environmental characteristics such as bright colours (*M* = 4, SD = 1.2), clinical feeling (*M* = 5.5, SD = 1.1), and high levels of organisation (*M* = 4.1, SD = 1.2) were rated non-favourably.

When asked to describe undesirable characteristics of an ineffective ASMR video that the participant had recently viewed, content analysis of qualitative responses (*N* = 115) revealed several common themes. About 27% of responses said that they did not find a trigger in the media that worked for them. Otherwise, the most often reported issue was obviously scripted content (27%). Participant comments include:

“The host had memorized a piece of tekst [sic] it was so blatantly obvious.”

“… too obvious asmr-triggering (not seamlessly interwoven in activity)”

“The host makes too many pop culture references, which I guess caters to her subscribers, but it ruins the mood for me, since it reminds me constantly that the situation is artificial and scripted.”

“Perhaps it seemed too much like a performance *for* me rather than say a role play exam *of* me.”

Auditory discomfort (16.5%) was also a common issue, with complaints centring around lack of quality sound mixing, causing trigger sounds to be too harsh. About 13% of comments also reported that the sound of the host’s voice being subjectively non-conducive to relaxation inhibited their ASMR.

Participants also complained that the video was ‘boring’ (4%) or ‘not relaxing’ (5%), with the following an example of the latter:

“The host was trashy and the whispering was very aggressive and included swearing.”

### Realistic content

Many qualitative contributions throughout the survey echo the sentiment that sounds created by objects in ASMR media should be realistic. For example:

“I like realistic sounds - immersive so that when I close my eyes it feels like I’m there.”

Though occasionally participants say that unexpected noises can cause a particularly strong tingling sensation, for the most part, participants emphasise that the soundscape should be representative of the noises viewers expect shown objects and environments to make.

### Distance from object

**Figure 2:**
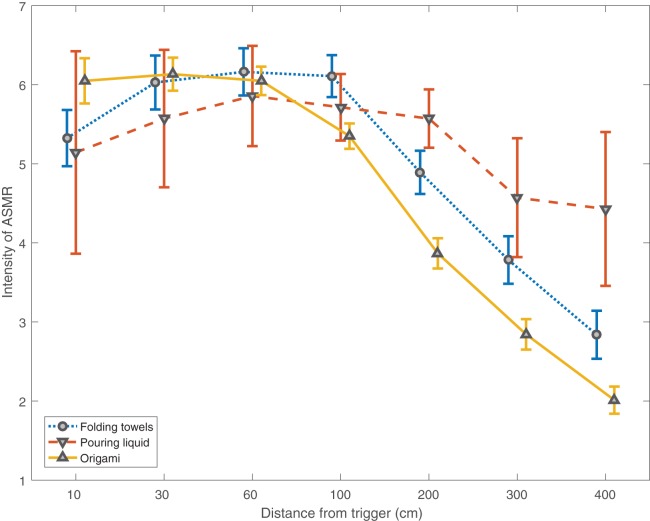
Intensity rating varies by distance. Average rating for anticipated intensity of ASMR tingles at varying distances from the trigger for three different sized trigger actions.

In order to better understand ideal camera angles for stimuli creation, participants were asked to rate the intensity of tingling sensations anticipated at a series of distances from an ASMR inducing activity. The sample (*N* = 127) rated smaller, more detailed actions, such as origami, to be best viewed from 60 cm or closer. Larger actions that can be better seen further away are best viewed from a distance of around 60 cm to 1 m away from the trigger object. Curiously, though potentially as small a visual stimulus as origami, the action of pouring liquid was rated as similarly effective up to a distance of 2 m from the trigger. These data are illustrated in Fig. 2.

### Visual aspects of trigger objects

When probed as to the factors which make an ASMR trigger object effective in online ASMR content (‘How important would the following factors be in triggering your ASMR?’), participants (*N* = 127) most strongly indicated that sounds made from the manipulation and use of objects was an important stimulus, with 51.2% of the sample rating it as ‘extremely important’. Visual aspects such as a focus on small details, the materials that trigger objects are made from, and symmetry receiving more mixed evaluations (see Table 1). Interestingly, the colour of ASMR trigger objects was rated as mostly unimportant in inducing ASMR, with 53.5% rating this factor as ‘not at all important’.

**Table 1:** Responses to the question ‘How important would the following factors be in triggering your ASMR?’ (*N* = 127).

	Extremely important (%)	Very important (%)	Moderately important (%)	Slightly important (%)	Not at all important (%)
Focus on small physical details	11	24.4	26.8	13.4	24.4
Focus on symmetry	7.1	14.2	24.4	15	39.4
The colour of the object	1.6	8.7	12.6	23.6	53.5
Focus on the material the object is made from	13.4	30.7	27.6	17.3	11
Sounds that the object would make if manipulated by the host	51.2	33.1	8.7	5.5	1.6

### Provision of context

Participants (*N* = 127) were prompted with an ASMR video scenario: ‘Imagine [object] has special historical significance. It was the first of its kind, and seeing it in use is very rare’. They were then asked to rate the following statement on a 1 (strongly disagree) to 5 (strongly agree) Likert scale: ‘Knowing [the above information] would increase the effectiveness of [an object] as an ASMR trigger’. A total of 127 participants responded in a mixed manner, 22% strongly disagreeing, 11% disagreeing, 22% neither agreeing nor disagreeing, 23% agreeing, and 11% strongly agreeing.

### Demonstration of trigger objects

In videos focusing on particular objects which are intended to trigger ASMR, hosts often manipulate the object without directly interacting with the viewer. In these cases, participants (*N* = 127) strongly favoured expert use of the object (*M* = 3.72, SD = 1.22) over a trial and error approach (*M* = 2.78, SD = 1.27) as shown by a two-tailed paired-samples *t*-test (*t*(124) = 6.71, *p* < 0.01).

### Audio

About 77% of the sample (*N* = 126) agreed with the statement ‘The pitch of the [trigger] sounds affects how strongly I feel tingles’. Only 10% disagreed with this statement. Further questions exploring whether high or low pitches were preferred revealed that lower pitches were rated as more likely to produce an intense tingling sensation; 12% of the sample agreed that higher-pitched trigger sounds caused more intense tingles (56% disagreed), and 56% agreed that lower-pitched trigger sounds produced more intense tingles (11% disagreed).

Of those participants who responded to a section probing hardware preferences (*N* = 108), 84% reported regularly consuming binaurally recorded ASMR media (Paul, 2009). About 58% said they feel binaural recording is more effective than regularly recorded audio for ASMR media consumption, whereas 16% disagreed with this sentiment. Further questioning revealed that 61% of the participants felt that binaural recording made the associated tingling sensation more intense.

Participants (*N* = 126) rated the statement ‘I only get tingles from sounds made by objects shown on the screen’ on a scale of one (strongly disagree) to seven (strongly agree). The results were somewhat split, with 31% of the sample agreeing somewhat to strongly, 65% disagreeing somewhat to strongly, and 4% neither agreeing nor disagreeing with the statement. Responses in the ‘disagree’ category favoured disagree (33%) and strongly disagree (22%).

When asked to rate the statement ‘Background music adds to how effective ASMR videos are at inducing tingles’ on a one (strongly disagree) to seven (strongly agree) Likert scale, participants heavily favoured disagree options. Only 14% of 126 respondents agreed to any extent, 10% neither agreed nor disagreed, and 72% disagreed to some extent. Of these disagree responses, strongly disagree (37%) and disagree (22%) were favoured.

These participants also rated the statement ‘Background music inhibits me from feeling strong tingles’ on the same seven point scale. Results were complimentary—71% of the sample agreed to some extent (with the option of strongly agree—28%—and agree—21%—favoured).

## Discussion

The results of the study provide a number of useful insights for those intending to create effective ASMR audio-visual stimuli for experimental use. Though responses both here and in other research (Barratt & Davis, 2015) make clear there is wide variety in effective stimuli between individuals, there appear to be several common threads in non-interpersonal ASMR trigger and viewing preferences. It is to be hoped that these results will be used to create lab and media content that is effective for the widest possible audience.

Previous research (Barratt & Davis, 2015) suggests that ASMR is a flow-like state (Csikszentmihalyi, 1979). As such, this study included some questions to probe whether or not participants identified their favourite ASMR content as having flow-like qualities which may aid the induction of such a state, and whether or not these qualities were universally required in order for content to be effective. The finding that participants prefer content that is happy, inviting, relaxed, and lacks danger, suggests that popular ASMR videos centring around the manipulation of objects may induce a flow-like state in viewers by depicting an environment that is conducive to flow. When viewed alongside consistent reports of obvious scripting resulting in ASMR videos being ineffective, and the strong preference for expert usage and manipulation of trigger objects over trial and error, this seems to suggest that participants are able to initiate their own experience of flow through viewing the effortless, automatic flow of movement that is associated with the state.

This idea of overall atmosphere being vital to ASMR induction is also supported by a large majority of participants choosing to skip those sections related to imagining achieving ASMR in a bar setting. This absence of response suggests that this type of environment, revealed in spontaneous reports from participants to be imagined as too loud, too public, and too chaotic, is extremely non-conducive to ASMR. These qualities stand in opposition to those associated with flow state, which may be why they are rated as undesirable when looking to achieve ASMR.

However, the participants did not rate their favourite ASMR content as predictable—this in itself may be evidence that this type of content does not necessarily need to depict all aspects of flow state in order to be effective, but rather to be pleasant and inviting enough to allow for relaxation. Further investigation is required in order to establish whether viewers take on a flow-like state from immersing themselves in the video, as one might expect through a sort of rubber-hand effect (Botvinick & Cohen, 1998; Ehrsson, Spence & Passingham, 2004), or rather find them relaxing enough to achieve that state independently of taking on depicted flow states.

The findings of the present study suggest a number of considerations for factors to control when creating ASMR inducing lab stimuli. Previous research (Barratt & Davis, 2015) indicates that ‘crisp’ sounds, such as the crinkling of tinfoil, are effective non-interpersonal ASMR triggers. However, viewed in isolation, this may paint a misrepresentative image of the types of sounds that are, in fact, capable of inducing ASMR. In particular, the results of the current study suggest that trigger sounds having a lower pitch are more frequently associated with intense ASMR sensations. Those sounds that are more complex, such as resonating tones, may also be especially appealing to viewers, as suggested by qualitative feedback.

Questions investigating physical attributes of triggers highlighted the fact that viewing small physical details of trigger objects, and exploring the material the trigger object is made of is somewhat important to the ASMR experience for the majority of respondents (see Visual aspects of trigger objects, above). Aspects such as colour and symmetry were less important to the induction of ASMR sensations. Participants responded most favourably, however, to the importance of sounds made by trigger objects. This suggests that visual aspects do have the ability to influence ASMR, but appear to be less vital to the experience than effective trigger sounds.

The interaction between sound and visuals displayed in ASMR videos is especially apparent in those results probing preferences of spatial distance from variously sized trigger object manipulations; Though the visual details of pouring liquid are intuitively better viewable from close range, the intensity of reported tingling sensations from viewing such an action remain high at distances less appropriate for viewing finer visual details. This result likely arose from the confounding factor of sound, the noises associated with pouring liquid being louder than those associated with origami or towel folding. As sounds can be appreciated from a further distance, louder stimuli remain effective regardless of the finer visual details being visible. This also suggests that auditory triggers may trigger ASMR more readily than visual triggers.

Providing additional contextual information about manipulated trigger objects may allow for a more intense ASMR experience for some viewers. Answers to questions probing this aspect were extremely mixed, with near equal support for ‘agree’ and ‘disagree’ options, and many remaining neutral. It seems that extra context to environments and triggers would enhance the viewing experience for some viewers.

In line with the relaxed, flow-like nature of ASMR videos, many tend to be slow paced, taking time to focus closely on trigger objects and sounds. The ideal length to focus on each trigger was rated as between 1 and 10 min. The spread of data in the current study seems to indicate that around 5–7 min would be palatable to the largest number of viewers. Nearly half of ASMR enthusiasts can comfortably focus on two triggers at once, but it may be wise to err on the side of caution, including only one at a time where possible.

The results suggest that sounds contained within ASMR content should be as realistic as possible, closely representing actual sounds that would be made by the object or material being manipulated. Ratings also suggested that the progression of the video and any actions or object manipulations shown should not feel forced, or appear overly scripted to the point of seeming unnatural. This is likely to be related to factors of flow state, as several results across both this and previous research (Barratt & Davis, 2015) suggest that an effortless, flow-like feel to content is conducive to ASMR induction.

Most respondents agreed that background music does not add to the experience of ASMR media, and in the majority of cases prevents the viewer from experiencing tingles. It is likely that background music would negatively affect the realism of the ASMR content, introducing a sense that it has been scripted, which was highlighted as a potentially off-putting feature in qualitative comments. Alternatively, background music may obscure trigger sounds, reducing their effectiveness. Therefore, background music should be avoided where possible when producing ASMR content.

For the majority of viewers, it seems that trigger sounds are effective without the object making the sound being shown on screen. As it is unlikely to be aversive to viewers to see the objects portrayed, however, those seeking to create ASMR stimuli may be wise to favour showing trigger objects on screen.

In this study, we demonstrate that several factors affect the experience of ASMR from media. Although ASMR triggers are highly variable between people, we found in general that videos should appear natural (unscripted); that sounds should be natural and lower-pitched, and that background music should not be used; mouth sounds should be avoided due to their divisiveness; that a focus on fine visual detail is effective; and that only a single trigger need be used.

It would potentially be advantageous in future research to devise a way to steer away from self-report data in order to more solidly establish the understanding of the sensory nature of ASMR, for example through covert motor or physiological monitoring. It is hoped that the current work may inform efforts to move in this direction.

## Supplemental Information

10.7717/peerj.3846/supp-1 Supplemental Information 1 Questionnaire text.

10.7717/peerj.3846/supp-2 Supplemental Information 2 Output of online questionnaire. The data here have been cleaned (incomplete responses removed) and anonymised. We also include marginal calculations for the benefit of the reviewers.

10.7717/peerj.3846/supp-3 Supplemental Information 3 Raw data. The dataset from the online questionnaire, stripped of potentially identifying information.

## Additional Information and Declarations

### Data Availability

The following information was supplied regarding data availability: The cleaned and anonymised output of the online questionnaire has been provided as a Supplemental File.
