# Supplementary material for: Sensory determinants of the autonomous sensory meridian response (ASMR): understanding the triggers
Source: PeerJ. 2017 Oct 6;5:e3846. doi: 10.7717/peerj.3846 (PMC5633022; doi:10.7717/peerj.3846)
Supplement: Supplemental Information 1 [file peerj-05-3846-s001.docx]

**Appendix 1: Full questionnaire**

[Page 1 includes demographic information]

**About your ASMR** (Page 2 of 12)

ASMR is a relaxed state in which individuals experience tingling, typically across the back of the head and shoulders, in response to certain stimuli. These stimuli often (but don't necessarily) include whispering, crisp noises (such as tinfoil), and watching others do simple, repetitive tasks. ASMR is not frisson (e.g. 'goosebumps' when listening to music)

Do you experience ASMR? [Yes/No]

Do you specifically search for and watch ASMR videos online? [Often/Sometimes/Never]

Do you specifically search for and listen to ASMR audios? [Often/Sometimes/Never]

Misophonia is a strong, negative emotional reaction to certain sounds. Trigger sounds for misophonia typically include non-verbal sounds made by the mouth, such as chewing and crunching.

Do you experience misophonia? [Yes/No/Unsure]

**Hosted ASMR content (Page 3 of 12)**

For this section, please recall one of your favourite ASMR videos that is hosted by a person/ASMRtist. Feel free to be as detailed as you like in your answers.

Is the video narrated? [Yes/No]

Where is the video set? [Indoors/Outdoors]

Is the setting private or public? [Private/Public]

Does the video contain roleplay, or a simulated situation (e.g. a haircut)? [Yes/No]

Is the video focused on an object? [Yes/No]

Briefly describe the contents of the video (e.g. "The host gives the viewer a haircut") [Comment box]

Please rate the appearance of the physical environment. [Variables: Clean, Intricate, Welcoming, Untidy, Bright Colours, Warm Looking, Modern. Scale: Strongly agree (1) to Strongly Disagree (7)]

Please briefly describe the physical setting of the video. [Comment box]

How would you rate the atmosphere of the video? [Variables: Happy, Inviting, Clinical, Organised, Relaxed, Predictable, Scripted, Dangerous. Scale: Strongly agree (1) to Strongly Disagree (7)]

Does the video have a solid colour background? [Yes, white/Yes, black/Yes, different colour/No]

What do you think makes this video most effective in helping you achieve ASMR? What makes it stand out above other ASMR videos? [Comment box]

When watching ASMR videos, how many triggers do you feel comfortable focusing on at one time? [1/2/3/4+]

Approximately how long, in minutes, is the whole video? [Comment box]

Approximately how long, in minutes, does the video spend focusing on each trigger? [Comment box]

Is there anything else about this video that you would like us to know? [Comment box]

**Non-hosted ASMR content (Page 4 of 12)**

For this section, please recall one of your favourite ASMR videos that does not focus on a host/ASMRtist. (e.g. natural scenes and sounds, slime videos, vacuuming)

Is the video narrated? [Yes/No]

Where is the video set? [Indoors/Outdoors]

Is the setting private or public? [Private/Public]

Does the video contain roleplay, or a simulated situation (e.g. a haircut)? [Yes/No]

Is the video focused on an object? [Yes/No]

Briefly describe the contents of the video (e.g. "The host gives the viewer a haircut") [Comment box]

Please rate the appearance of the physical environment. [Variables: Clean, Intricate, Welcoming, Untidy, Bright Colours, Warm Looking, Modern. Scale: Strongly agree (1) to Strongly Disagree (7)]

Please briefly describe the physical setting of the video. [Comment box]

How would you rate the atmosphere of the video? [Variables: Happy, Inviting, Clinical, Organised, Relaxed, Predictable, Scripted, Dangerous. Scale: Strongly agree (1) to Strongly Disagree (7)]

Does the video have a solid colour background? [Yes, white/Yes, black/Yes, different colour/No]

What do you think makes this video most effective in helping you achieve ASMR? What makes it stand out above other ASMR videos? [Comment box]

When watching ASMR videos, how many triggers do you feel comfortable focusing on at one time? [1/2/3/4+]

Approximately how long, in minutes, is the whole video? [Comment box]

Approximately how long, in minutes, does the video spend focusing on each trigger? [Comment box]

Is there anything else about this video that you would like us to know? [Comment box]

**Non-effective ASMR content (Page 5 of 12)**

For this section, please recall an ASMR video that you thought would be effective at triggering ASMR, but wasn’t.

Is the video narrated? [Yes/No]

Where is the video set? [Indoors/Outdoors]

Is the setting private or public? [Private/Public]

Does the video contain roleplay, or a simulated situation (e.g. a haircut)? [Yes/No]

Is the video focused on an object? [Yes/No]

Briefly describe the contents of the video (e.g. "The host gives the viewer a haircut") [Comment box]

Please rate the appearance of the physical environment. [Variables: Clean, Intricate, Welcoming, Untidy, Bright Colours, Warm Looking, Modern. Scale: Strongly agree (1) to Strongly Disagree (7)]

Please briefly describe the physical setting of the video. [Comment box]

How would you rate the atmosphere of the video? [Variables: Happy, Inviting, Clinical, Organised, Relaxed, Predictable, Scripted, Dangerous. Scale: Strongly agree (1) to Strongly Disagree (7)]

Does the video have a solid colour background? [Yes, white/Yes, black/Yes, different colour/No]

What do you think makes this video most effective in helping you achieve ASMR? What makes it stand out above other ASMR videos? [Comment box]

When watching ASMR videos, how many triggers do you feel comfortable focusing on at one time? [1/2/3/4+]

Approximately how long, in minutes, is the whole video? [Comment box]

Approximately how long, in minutes, does the video spend focusing on each trigger? [Comment box]

Is there anything else about this video that you would like us to know? [Comment box]

**Offline ASMR experiences (Page 6 of 12)**

These questions focus on the environments in which ASMR might occur offline. When answering, please think about the overall structure of spaces in which you have experienced ASMR, as well as specific triggers.

Have you ever experienced ASMR in a public place? [Yes/No]

Have you experienced ASMR in an open, natural space? (briefly describe) [Comment box]

Have you experienced ASMR in an enclosed, natural space? (briefly describe) [Comment box]

Have you experienced ASMR in larger, open man-made space? (briefly describe) [Comment box]

Have you experienced ASMR in a smaller, enclosed man-made space? (briefly describe) [Comment box]

**ASMR in specific situations (Page 7 of 12)**

These questions ask you about ASMR in specific situations.

Have you ever experienced ASMR in a bar? [Yes/No]

If yes, please rate the appearance of the physical environment in the bar. [Variables: Clean, Intricate, Welcoming, Untidy, Bright Colours, Warm Looking, Modern. Scale: Strongly agree (1) to Strongly Disagree (7)]

If yes, how would you rate the atmosphere of the bar? [Variables: Happy, Inviting, Clinical, Organised, Relaxed, Predictable, Scripted, Dangerous. Scale: Strongly agree (1) to Strongly Disagree (7)]

Imagine for a moment that you are creating an ASMR video in a bar. You are looking for triggers that your viewers would most enjoy. Which of the following do you think would make the best triggers? Please select your favourite 4. [Options: The pouring of drinks/watching the staff at work/The Buzz of friendly conversations/Glasses and ice ‘chinking’/The order of objects behind the bar/Focus on the material the bar itself is made from/Watching bubbles in fizzy drinks/the layout of the bar.]

Are there any other triggers that would be found in a bar scene that you would include in your video? [Comment box]

How familiar are you with the Scottish countryside? [Scale: Extremely familiar (1) to Not At All Familiar (5)]

Imagine for a moment you were creating an ASMR video in the Scottish countryside. Briefly describe the kind of countryside where you could make this video. [Comment box]

You are looking for triggers that you think your viewers would enjoy. Which of the following would make the best triggers? Please select your top four. [Options: Wide shots of the countryside/Close ups and sounds of local plants/Close ups and sounds of local wildlife/Close ups and sounds of natural stone textures/ Close ups and sounds of natural wood textures/Wide shots of running water/Wide shots of locals walking through fields/Shots of swirling clouds above/The sound of the wind]

Please use this box to elaborate on your above selections. (eg. Specific types of terrain, textures) [Comment box]

Are there any other Scottish triggers you would like to include in your video? If so, which?

[Comment box]

Please rate the appearance of the environment in the video about the Scottish countryside. [Variables: Clean, Intricate, Welcoming, Untidy, Bright Colours, Warm Looking, Modern. Scale: Strongly agree (1) to Strongly Disagree (7)]

**Sounds in ASMR videos (Page 8 of 12)**

These questions relate to sound in ASMR videos

Please rate the following statements according to how true each is for you when watching ASMR videos


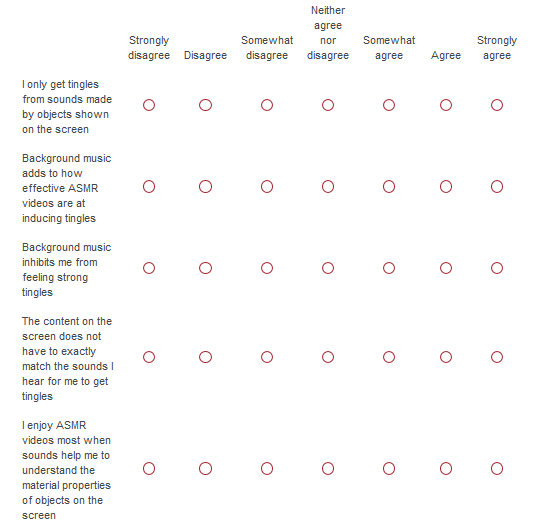


Please rate the following statements according to how true each is for you when listening to ASMR content (either as audio alone, or as part of a video)


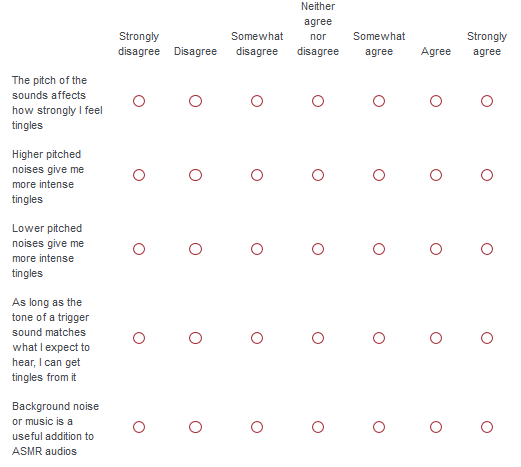


**Congruent sounds in ASMR (Page 9 of 12)**

Imagine that you are watching an ASMR video in which a host is tapping on the side of a large ceramic bowl. Around half way though the video, the sound of the tapping changes. Instead of the sound of a bowl being tapped, you hear the sound of someone tapping the handle of a wooden hairbrush.

Please select one of the following: [The unexpected sound increases the intensity of my tingles/The unexpected sound decreases the intensity of my tingles/The unexpected sound stops my tingles/The unexpected sound has no effect on my tingles.]

Please rate the following statements:


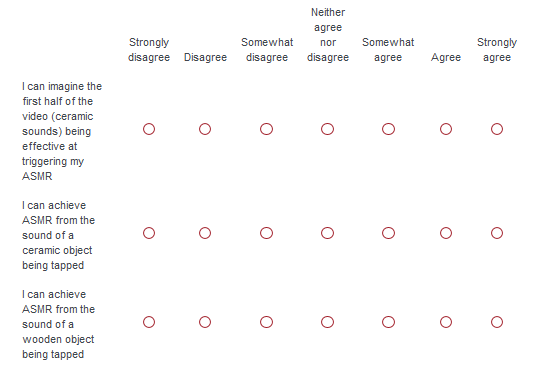


Imagine that you are watching an ASMR video in which a host is stacking up a four of small metal building blocks. The edges of the blocks align perfectly. As the host stacks, you can hear each block tap against the one below it. As the host places the last block, they twist their hand slightly and place it crookedly, the edges no longer in line with the block below it.

Please select one of the following: [The unexpected placement increases the intensity of my tingles/The unexpected placement decreases the intensity of my tingles/The unexpected placement stops my tingles/The unexpected placement has no effect on my tingles.]

Please rate the following statements:


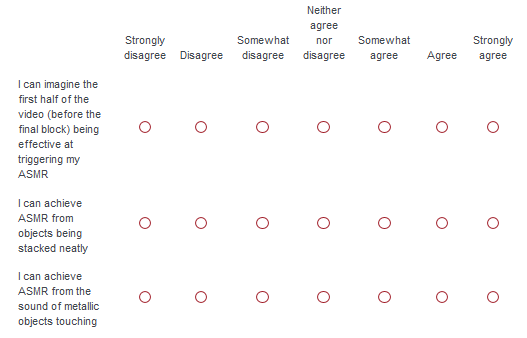


**Distance from the action (Page 10 of 12)**

Imagine that an ASMR host is creating a live ASMR experience. They have done a great job creating a comfortable space for viewers to relax in. They give you several options for things you could watch them do.

Which action would you most like to watch?[Folding towels/Pouring liquid into a glass/Folding origami]

What would you enjoy most about your chosen action? [Comment box]

Would your distance from the host while they perform affect how strongly you experience tingles? [Yes/Unsure/No]

Imagine you are invisible in this situation. How close would you like to be seated to the host? [Comment box]

Imagine you are watching the host manipulate your chosen object from the following distances. Please rate them according to how intense your tingles would be at each distance


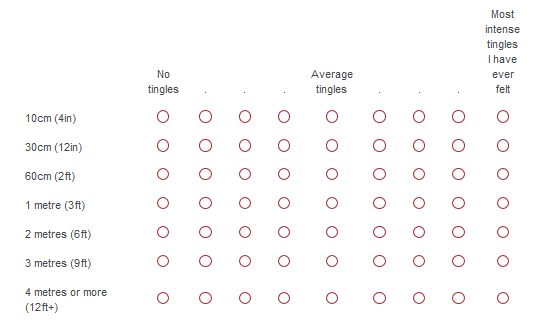


**Object interactions (Page 11 of 12)**

Imagine you are watching an ASMR video which focuses on one small object, resting on a table. You have never seen this object before, and can only guess at what it might be for.


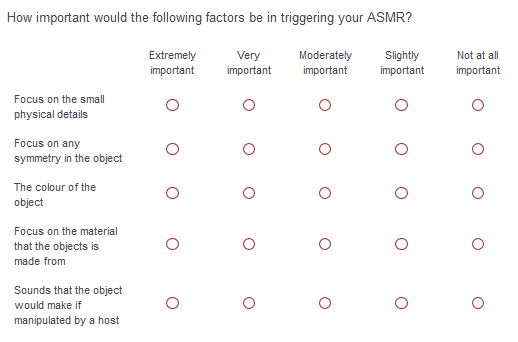


Imagine this object has special historical significance. It was the first of its kind, and seeing it in use is very rare


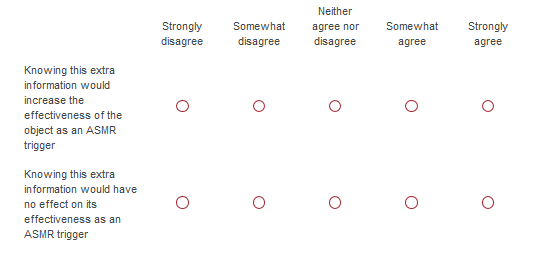


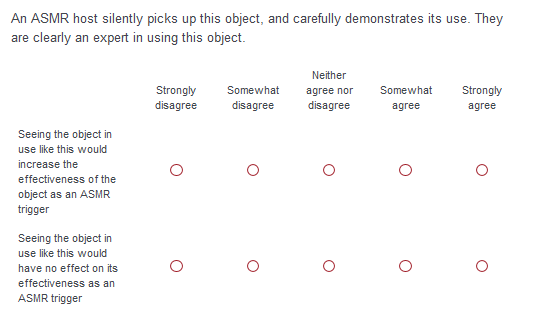


Alternatively, an ASMR host silently picks up this object, and works out how to use it through trial and error. It is clear they have never used it before.


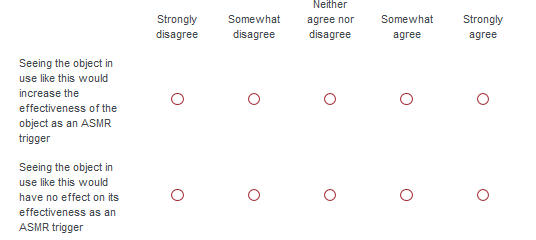


**About your hardware (Page 12 of 12)**

These questions ask you about the hardware you use to consume ASMR media


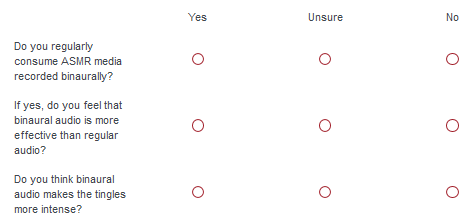


What hardware do you use to watch ASMR videos? Tick all that apply. [Desktop/Laptop/Smartphone/Tablet/TV/other]

Which of the below do you feel is most effective for viewing ASMR videos? Select one option.

[Desktop/Laptop/Smartphone/Tablet/TV/other]

Do you use headphones when viewing ASMR videos? [Scale: Never (1) to Always (5)]

Do you ever find yourself adjusting the settings of your screen for ASMR videos? [Yes/No]

Please rate the following statements


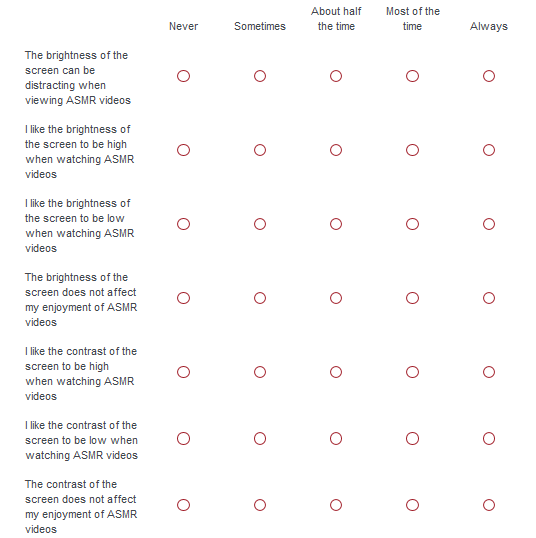


The following questions are about 'haptic feedback', such as the vibration capabilities of a mobile phone. Please rate the following:


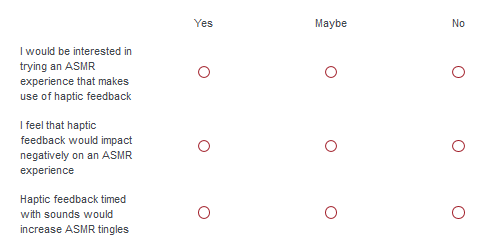


How would you imagine that haptic feedback would best be included in an ASMR experience? [Comment box]
